# Response of C2C12 Myoblasts to Hypoxia: The Relative Roles of Glucose and Oxygen in Adaptive Cellular Metabolism

**DOI:** 10.1155/2013/326346

**Published:** 2013-11-05

**Authors:** Wei Li, Zhen-Fu Hu, Bin Chen, Guo-Xin Ni

**Affiliations:** ^1^Clinical Medicine Education Center, Nanfang Hospital, Southern Medical University, Guangzhou 510515, China; ^2^Department of Plastic Surgery, Nanfang Hospital, Southern Medical University, Guangzhou 510515, China; ^3^Department of Orthopaedics and Traumatology, Nanfang Hospital, Southern Medical University, Guangzhou 510515, China

## Abstract

*Background*. Oxygen and glucose are two important nutrients for mammalian cell function. In this study, the effect of glucose and oxygen concentrations on C2C12 cellular metabolism was characterized with an emphasis on detecting whether cells show oxygen conformance (OC) in response to hypoxia. *Methods*. After C2C12 cells being cultured in the levels of glucose at 0.6 mM (LG), 5.6 mM (MG), or 23.3 mM(HG) under normoxic or hypoxic (1% oxygen) condition, cellular oxygen consumption, glucose consumption, lactate production, and metabolic status were determined. Short-term oxygen consumption was measured with a novel oxygen biosensor technique. Longer-term measurements were performed with standard glucose, lactate, and cell metabolism assays. *Results*. It was found that oxygen depletion in normoxia is dependent on the glucose concentration in the medium. Cellular glucose uptake and lactate production increased significantly in hypoxia than those in normoxia. In hypoxia the cellular response to the level of glucose was different to that in normoxia. The metabolic activities decreased while glucose concentration increased in normoxia, while in hypoxia, metabolic activity was reduced in LG and MG, but unchanged in HG condition. The OC phenomenon was not observed in the present study. *Conclusions*. Our findings suggested that a combination of low oxygen and low glucose damages the viability of C2C12 cells more seriously than low oxygen alone. In addition, when there is sufficient glucose, C2C12 cells will respond to hypoxia by upregulating anaerobic respiration, as shown by lactate production.

## 1. Background

Oxygen and glucose are crucial for cellular respiration. Glycolysis underlies both anaerobic and aerobic respiration, but aerobic respiration provides a greater yield of ATP per molecule of glucose if oxygen is available. The ability of a mammalian cell to gauge the oxygen concentration in its environment and to protect itself through internal regulation is known as oxygen sensing [1, 2]. Normal cellular function depends on the supply of oxygen, that is, sufficient for metabolic needs. If the oxygen supply does not match the cellular energy demands, adaptive mechanisms would occur to compensate. However, once such adaptive mechanisms are insufficient to prevent cellular hypoxia, an increase in anaerobic synthesis of ATP would be expected to meet the demand [3]. Under anoxic condition, the rate of glycolysis in mammalian cells is considered to be insufficient to maintain intracellular concentrations of ATP.

The conventional view that a decrease in oxygen consumption will only be caused by oxygen limitation at mitochondria is now challenged by a phenomenon described as oxygen conformance (OC), by which oxygen consumption declines as the oxygen supply is reduced without either a loss of cellular viability or compensatory anaerobic energy production [4, 5]. OC has been reported in hepatocytes, skeletal muscle, and cardiomyocytes [6–9]. In these cell types, oxygen consumption under condition of low oxygen concentration is reduced to maintain cellular survival without an increase in anaerobic energy production. 

C2C12 murine myoblast has been widely applied to *in vitro* studies on skeletal muscle damage, regeneration, and differentiation, as well as chemical, electrical and mechanical stimulation due to the cell line's rapid growth, ease of culture, and sensitivity to manipulations [10–18]. In addition, it has been extensively used for the production of 3D tissue-engineered muscle [19–24]. Previously, it was declared that the C2C12 cell line exhibited OC behavior [25]. More recently, Gawlitta et al. [26] investigated how the metabolism, including glucose utilization, of C2C12-engineered skeletal muscle is influenced by ischemic factors and suggested that during hypoxia, anaerobic metabolism is adopted, resulting in lactic acid accumulation, and cellular metabolism is downregulated by the occurrence of OC. However, in these studies, the OC rate of C2C12 cell, which is a key parameter to verify the occurrence of OC, was not determined. Therefore, to further understand the response of myoblast to hypoxia, in the present study, the aerobic and anaerobic respirations in C2C12 murine skeletal myoblast were investigated by measuring oxygen consumption, glucose consumption, lactate production, and metabolic activity in different glucose concentrations and under conditions of normoxia and hypoxia.

## 2. Material and Methods

### 2.1. Cell Culture

C2C12 murine skeletal muscle myoblasts (passages 15–30, ECACC, Salisbury, UK) were cultured in tissue culture flasks (Sarstedt), in a humidified, 37°C, 5% CO_2_ incubator and passaged every 2-3 days at 80–90% confluency. Growth media (GM) for cell propagation was DMEM (4.5 g/L glucose, containing GlutaMAX) to which sterile supplements were added as follows (per 100 mL): 10 mL FCS (gamma-irradiated, containing 1.16 g/L glucose; BioSera), 1 mL sodium pyruvate (100 mM), 0.5 mL Penicillin (10,000 U/mL), streptomycin (10,000 ug/mL), and 1 mL uridine. 

For all experiments, media were changed to experiment media (EM), which was phenol red-free DMEM (D5030, Sigma, plus 3.7 g/L sodium bicarbonate), supplemented as GM but with the addition of 2 mL glutamine (200 mM) and the omission of uridine. Glucose was added to EM prior to supplementation at 0 g/L (low glucose, LG), 1 g/L (medium glucose, MG), and 4.5 g/L (high glucose, HG). Due to the presence of glucose in the supplemental FCS, the final glucose concentration in experiment media was 0.6 (LG), 5.6 (MG), and 23.3 (HG) mM, respectively. EM were equilibrated overnight in a standard 5% CO_2_ incubator at 37°C before being added to cells. Tissue culture reagents were purchased from Invitrogen unless otherwise stated; chemicals were purchased from Sigma unless otherwise stated.

### 2.2. Oxygen Measurement Using BD Biosensor System

Two hundred microlitre aliquots of cell suspensions, at 3 densities of 50,000, 100,000 and 200,000 cells/mL, swere transferred into 96-well oxygen biosensors (Becton Dickinson Bioscience, Oxford, UK). The loaded biosensors were transferred immediately to the 5% CO_2_ humidified environment of a standard cell culture incubator referred to the normoxic condition in this study. As for hypoxia condition, biosensors loaded as above were transferred into a humidified incubator (Innova CO-48) set to maintain a 1% O_2_, 5% CO_2_, 37°C environment. The oxygen concentration of the samples was recorded from the base of the wells by scanning the biosensor plate using a fluorescence plate reader (Fluoroskan Ascent FL,Thermo Lab systems) at hourly intervals over a 6-hour period. 

### 2.3. Biosensor System Calculations

The oxygen biosensor (BD Biosciences, Oxford, UK) consists of a 96-well plate with an oxygen sensitive dye embedded within a gas permeable silicon matrix immobilized at the base of each well. Oxygen quenches the ability of the dye to fluoresce in a concentration-dependent manner. The fluorescence intensity at the base of each well was read using excitation and emission filters of 485 and 595 nm, respectively. The quenching behavior of the biosensor fluorophores is described by a mathematical model, namely, the Stern-Volmer equation:

(1) $$\begin{array}{c} \frac{I_{0}}{I}=1+K_{\text{sv}}\times \left[{\text{O}}_{2}\right], \end{array}$$

where [O_2_] is the oxygen concentration, *K*_sv_ is the fluorophore quenching constant, *I* is the normalized fluorescence intensity at [O_2_], and *I*_0_ is the normalized fluorescence intensity recorded at the reference condition (zero oxygen). The value of *K*_sv_ can be calculated from the ratio of the fluorescence intensity recorded from two reference conditions. In the present work, these correspond to a cell-free medium sample that had equilibrated with the incubator atmosphere, referred to as the ambient control [O_2_]_*A*_ and a zero oxygen control that consisted of 100 mM sodium sulphite, such that

(2) $$\begin{array}{c} K_{\text{sv}}=\frac{I_{0}/I_{A}-1}{{\left[{\text{O}}_{2}\right]}_{A}}, \end{array}$$

where *I*_0_ is the normalized fluorescence intensity in the zero oxygen control and *I*_*A*_ is the normalized fluorescence intensity a [O_2_]_*A*_. The values *I*_0_ and *I*_*A*_ were measured using the biosensor, where the value of [O_2_]_*A*_ was calculated using Henry's law for the equilibrium distribution of oxygen between the atmosphere and water, and defined as (3)

(3) $$\begin{array}{c} {\left[{\text{O}}_{2}\right]}_{A}=\frac{\alpha }{V_{m}}\times {\text{PO}}_{2}, \end{array}$$

where [O_2_]_*A*_ is the concentration of oxygen in solution [moles/L], *α* is the solubility coefficient of oxygen expressed as volume of gas per volume of water, *V*_*m*_ represents the molar volume of oxygen at standard temperature (0°C) and pressure (1 Atm), and PO_2_ is the partial pressure of oxygen in the atmosphere. According to Avogadro's theory, the solubility coefficient of oxygen in water is 0.024 and the value of *V*_*m*_ for oxygen is 22.4 L at 37°C. The partial pressure of oxygen in air at sea level (0.210 atm) is reduced to 0.186 atm under the test conditions by the additional CO_2_ (0.050 atm) and water vapor (0.062 atm) in the incubator atmosphere. Substituting these values into (3) yields the oxygen concentration in the ambient control of 200 *μ*M and in the 1% oxygen CO_2_ incubator control of 10.7 *μ*M [27].

### 2.4. Glucose and Lactate Measurement

For each cell density supplied with HG medium, additional replicates were prepared as above but in standard separate 96-well plates and cultured for up to 72 hours to provide samples for glucose and lactate determinations. The medium was aspirated at 24-hour intervals, transferred immediately to prelabeled micro-centrifuge tubes on ice, and then frozen prior to subsequent determination of the glucose and lactate concentrations, as below.

Standard protocols for glucose and lactate measurements were followed. In brief, glucose concentrations in the medium were determined using the infinity glucose assay reagent (Sigma Diagnostics). Infinity reagent contains hexokinase, glucose-6-phosphate dehydrogenase, and NAD^+^. 240 *μ*L of reagent was added to 12 *μ*L aliquots of each diluted sample, preculture media controls, or glucose standard solutions in duplicate and the reactions were incubated at 37°C for 10 minutes before recording the absorbance at 340 nm using a plate reader. For lactate measurements, 10 mg of *β*NAD^+^ was reconstituted with 2 mL glycine buffer, 4 mL water, and 0.1 mL LDH suspension. 140 *μ*L of the reconstituted *β*NAD^+^ was added to 6 *μ*L of sample, lactate standards, sand pre-culture media controls in duplicate and incubated at 37°C for 30 minutes before the absorbance was recorded at 340 nm. Samples that exceeded the standard curve were diluted and remeasured.

### 2.5. Alamar Blue Assay

The Alamar Blue assay is a commercially available product (Serotec Ltd., Oxford, UK) as an indicator of cellular metabolic status. For each of the three cell densities, additional replicates were prepared as above in standard separate 96-well plates and cultured for up to 48 hours. Alamar Blue reagent was added to cell culture media at a concentration of 10% (v/v) then incubated in a standard 5% CO_2_ incubator at 37°C for 4 hours then fluorescence was measured (excitation wavelength 570 nm and emission wavelength at 590 nm, Fluoroskan Ascent FL; Thermo Lab systems).

### 2.6. Statistical Analysis

All data are presented as mean ± standard deviation. The effect of different glucose concentrations (LG, MG, and HG) and of the cultivation environment (either normoxic or hypoxic) on the metabolic status of C2C12 cells was analyzed simultaneously by one-way analysis of variance (ANOVA) performed with GraphPad Prism version 5. Individual comparisons were subsequently made between each glucose concentration using a Bonferroni post tests. Statistical significance is indicated as *P* > 0.05 (non-significant, ns); *P* < 0.05 (*); *P* < 0.01 (**); and *P* < 0.001 (***).

## 3. Results

### 3.1. Effect of Glucose Concentration on Oxygen Consumption of C2C12 Cells

**Figure 1:**
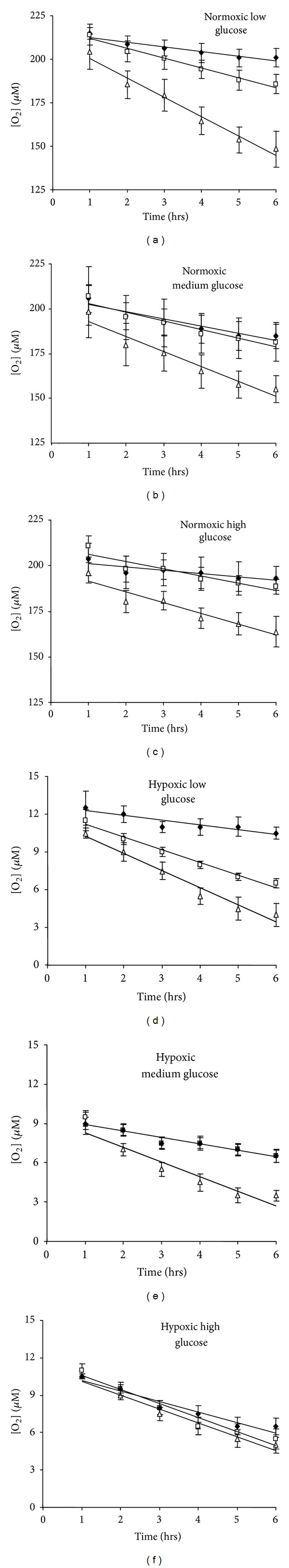
The depletion of oxygen within BD biosensor system containing C2C12 cells over a 6-hour culture period. The figures compare culture in low glucose (LG), medium glucose (MG), and high glucose (HG). The results are shown for 200 *μ*L cell samples of 0.5 × 10^5^ cells/mL (♦), 1 × 10^5^ cells/mL (□), and 2 × 10^5^ cells/mL (Δ) cultured with the appropriate medium exposed in normoxic ((a)–(c)) and hypoxic ((d)–(f)) environments. Each point represents the mean and standard deviation of six replicates.

**Figure 2:**
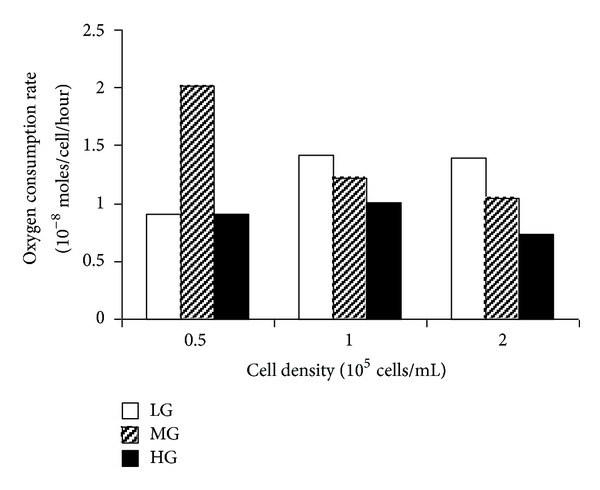
The initial oxygen consumption rate of C2C12 cells cultured in LG, MG, and HG media at normoxic environment. Values are estimated from the linear region of the oxygen depletion curve, as indicated in Figures 1(a)–1(f).

Figures 1 and 2 showed oxygen consumption of C2C12 cells of various densities cultured with different glucose concentrations under either normoxic or hypoxic condition. Under normoxic condition, a monotonic reduction in oxygen concentration was observed over six hours, and this oxygen consumption was proportional to cell density for cells cultured at LG level (Figure 1(a)). At either MG or HG level, the oxygen consumption was similar for cells cultured at densities of 0.5 × 10^5^ and 1 × 10^5^ cells/mL but higher than that at a density of 2 × 10^5^ cells/mL (Figures 1(b) and 1(c)). The highest oxygen consumption was observed with cells cultured in LG conditions at a density of 2 × 10^5^ cells/mL.

Under normoxic condition, a linear model was fitted for each experimental variable to the data from 1 to 6 hrs. OC rate was determined from the gradient of this line and converted to oxygen moles/hour/cell (Figure 2). The rate did not appear to be substantially affected by the cell density; however, when comparing cells cultured at density of 1× and 2 × 10^5^ cells/mL, it was found that OC rate decreased with the increase glucose concentration.

Figures 1(d)–1(f) presented data from experiments concerning hypoxia where the biosensor plates were cultured in conditions of 1% oxygen but transferred to the 21% oxygen environment of room air at hourly interval for fluorescence measurement. The measurement procedure took around 3 minutes during which the biosensor plates were equilibrating with 21% oxygen instead of 1% oxygen. Consequently, the recorded fluorescence should be normalized to an intermediate value between 1% (incubation condition) and 21% (measurement condition), which is not precisely known. Due to this technical limitation, the oxygen concentrations shown in the *y*-axis labels were somewhat arbitrary (Figures 1(d)–1(f)) and further analysis, such as calculation of oxygen consumption rates, was therefore not attempted. However, trends may still be identified from these data; for example, Figure 1(d) revealed clear differences between cells cultured at different densities, which are likely to be attributed to oxygen consumption by the cells. A similar relationship was suggested to that observed in normoxia between oxygen concentration, cell density, and glucose concentration (Figures 1(d)–1(f)).

### 3.2. Effect of Hypoxia on Glucose Uptake and Lactate Production of C2C12 Cells

**Figure 3:**
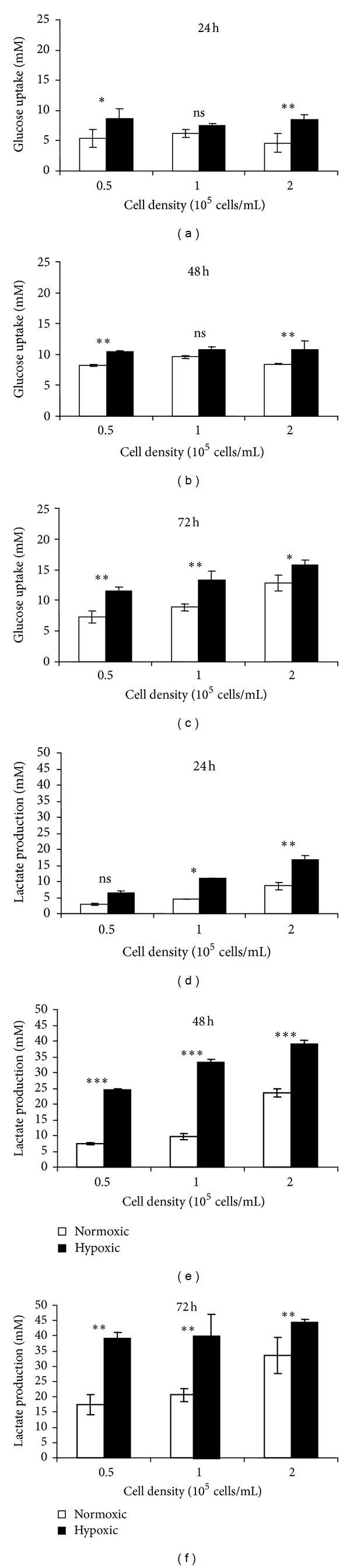
Glucose uptake ((a)–(c)) and lactate production ((d)–(f)) of C2C12 cells cultured in HG medium at hypoxic and normoxic environments up to 72 hours period. Each bar represents the mean and standard deviation of six replicates. Comparisons of quantitative significant differences are shown in the figure, significance is indicated as nonsignificant, *P* > 0.05 (ns), *P* < 0.05 (*), *P* < 0.01 (**), and *P* < 0.001 (***).

Glucose uptake and lactate production were measured for cells cultured at three different densities in normoxia or hypoxia for 24, 48, and 72 hours (Figure 3). The effect of supplemental glucose was not examined and all cells were cultured in HG conditions.

In normoxia, glucose uptake was similar for three cell densities at 24 and 48 hrs (Figures 3(a) and 3(b)) but was clearly proportional to cell density by 72 hrs (Figure 3(c)). Lactate production was proportional to cell density at 24, 48 and 72 hours (Figures 3(d)–3(f)). In hypoxia, the same patterns were seen as for normoxia; however, both glucose uptake and lactate production were consistently higher in hypoxia than in normoxia. Glucose uptake and lactate production increased sharply over the three day-time course of the experiment, and such increase was greater in hypoxia than that in normoxia (Figure 3).

### 3.3. Effect of Glucose Concentration and Hypoxia on the Metabolic Activity of C2C12 Cells

**Figure 4:**
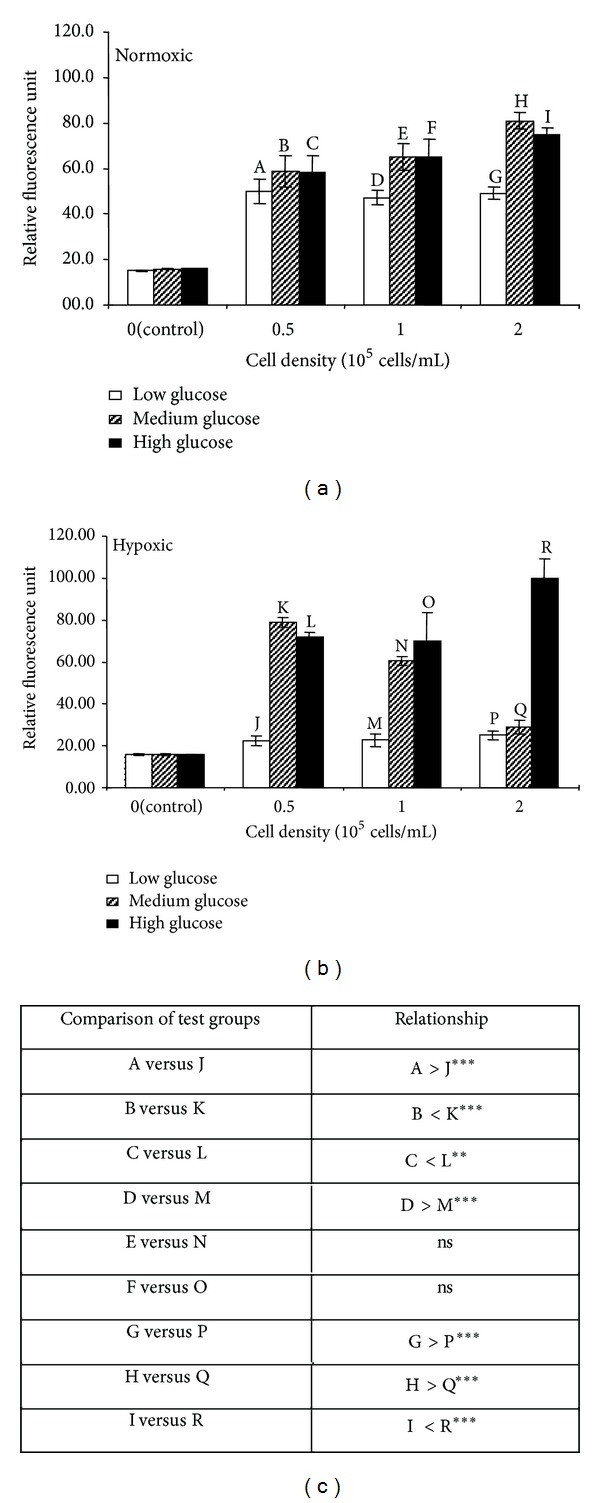
Alamar Blue assay. Cellular metabolic states of C2C12 cells cultured with LG, MG, and HG media exposed at normoxic (a) and hypoxic (b) environment for 48 hours. Each bar represents the mean and standard deviation of eight replicates. Comparisons of quantitative significant differences are shown in the table (c) significance is indicated as *P* < 0.05 (*), *P* < 0.01 (**), *P* < 0.001 (***), and nonsignificant, *P* > 0.05 (ns).

**Figure 5:**
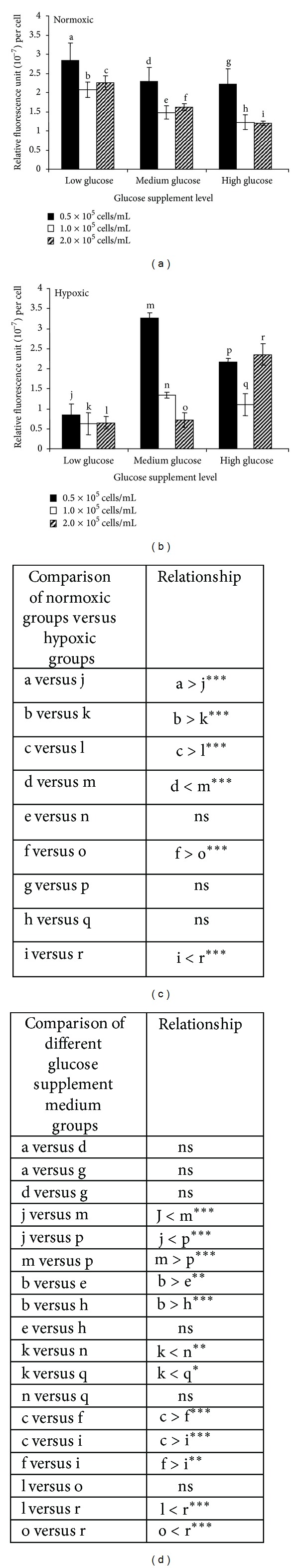
Cellular metabolic state of each C2C12 cell in different cell densities (0.5 × 10^5^ cells/mL, 1 × 10^5^ cells/mL and 2 × 10^5^ cells/mL), and in different glucose supplement media (LG, MG and HG) culture exposed at normoxic (a) and hypoxic (b) environments for 48 hours. Comparisons of quantitative significant differences are shown in the table (c) significance is indicated as *P* < 0.05 (*), *P* < 0.01 (**), *P* < 0.001 (***), and nonsignificant, *P* > 0.05 (ns).

Alamar Blue assay was used to investigate the metabolic activity of C2C12 cells after culture for 48 hrs under different glucose and oxygen conditions (Figures 4 and 5). In Alamar Blue assay, a nonfluorescent substrate is converted to a fluorescent product by the activity of cellular enzymes. However, when this assay is used for cells growing in culture for long periods of time (days), an effect that causes an increase in cell number cannot be distinguished from an effect that increases individual cell activity (in both cases the fluorescent output will be increased); consequently, we carried out cell counts in parallel and attempted to normalise Alamar Blue assay results to contemporaneous cell counts (rather than the initial seeding density).

In normoxia, fluorescence produced in Alamar Blue assay was similar for all three densities of cells cultured in the LG condition but was proportional to cell density for cells cultured in either MH or HG condition (Figure 4(a)). Comparing cells grown at the same density but in different glucose concentrations, fluorescence tended to increase as the glucose concentration increased. Under hypoxia, the fluorescence generated by cells grown in LG condition was significantly lower than the equivalent cells in normoxia (Figure 4(c)). Cells grown in MG condition showed similar level of fluorescence in hypoxia and normoxia, whilst cells grown in HG condition showed greater level of fluorescence in hypoxia than that in normoxia (especially clear for cell density of 2 × 10^5^ cells in high glucose, Figures 4(a) and 4(b)). As for hypoxia condition, the greatest difference was seen between cells at a density of 0.5 × 10^5^ cells/mL where the normalized fluorescence for cells in MG condition was twofold higher than that in LG condition.

The effect of normalizing the Alamar Blue data presented in Figure 4 to contemporaneous cell counts was shown in Figure 5. In normoxia, there was a trend for the activity per cell to decrease as the glucose concentration increased, and this trend was most clear at densities of 1 and 2 × 10^5^ cells/mL (Figures 5(a) and 5(d)). This suggested that the overall increase in fluorescence that occurs with the increase in glucose concentration before normalisation (Figure 4(a)), is due to an increase in cell number rather than an increase in individual cell activity. Indeed there is a suggestion that increasing glucose decreases individual cell activity. On the other hand, in hypoxia, activity per cell in low glucose condition was reduced compared to normoxia, whilst activities were similar for cells in medium and high glucose. Activity per cell tended to increase as the glucose concentration increased (Figure 5(b)), which is opposite to that found in normoxia (Figure 5(a)). It was supposed that under hypoxia the increase in fluorescence as glucose increased may be due to both an increase in cell number and an increase in the activity of each cell.

## 4. Discussion

The aim of this study was to understand the effect of glucose concentration and oxygen supply on oxygen depletion, glucose uptake, lactate production, and metabolic activity in C2C12 cells, a murine skeletal myoblast cell line. It was found that, firstly, the rate of oxygen depletion was dependent on the glucose concentration in the culture medium in normoxia. Secondly, glucose uptake and lactate production increased in hypoxia; thirdly, with the increase in glucose concentration, metabolic activity decreased in normoxia, but increased in hypoxia; and finally, metabolic activity was reduced by hypoxia in LG and MG conditions, but unchanged in HG condition. 

Arthur et al. [25] ever investigated the effect of hypoxia (5 *μ*M oxygen) on C2C12 cells and reported that this cell line responds to hypoxia by showing OC. However, our data did not support the occurrence of OC. Such disparity may be attributed to differences in study design between these two studies. Firstly, the time course selected in their study was 0–2 hours, whereas we examined the responses for up to 72 hours, which should be more relevant to the general culture situation. Secondly, they employed a perfusion system to better control oxygen level but, unfortunately, created a condition, that is, not close to the normal culture situation, thus leading to a 10–20% loss of cell viability even over 1-2 hours. It is quite possible that OC is an acute cell response to hypoxia, which does not persist over time. Alternatively, it is the gradual reduction (as applied by Arthur et al. [25]) not the step-change in oxygen level (employed in present study) that triggers OC. Contrary to this earlier study, our findings suggest that metabolic responses to hypoxia are greatly affected by glucose concentrations rather than oxygen availability.

Our findings demonstrated that C2C12 cells respond to long-term hypoxia by increasing glucose consumption, which indicates an increase in anaerobic respiration. OC was not observed in present study, which suggested that when there is sufficient glucose, C2C12 cells will respond to hypoxia by up-regulating anaerobic respirations, as shown by lactate production. Metabolic activity was reduced when hypoxia was encountered in MG or LG but not reduced when cells experienced hypoxia in the presence of HG, suggesting that a combination of low oxygen and low glucose damaged the viability of C2C12 cells more seriously than low oxygen alone. Although cell density was shown to affect the magnitude of measured values, in most experiments, the same trend was observed at all three cell densities, suggesting that these results are not an artifact of the particular cell density studied.

In the present study, the oxygen consumption rate of C2C12 cells in normoxia was around 3 × 10^−13^ moles oxygen/hour/cell. This equates to an oxygen utilization rate (OUR) of 5 fmole min^−1^ cell^−1^, which was similar to those reported by Guarino et al. on a number of cell types [28]. Our results showed that oxygen consumption decreased as glucose concentration increased. There was an upregulation in oxygen consumption rate of cells cultured in LG and MG compared to cells cultured in HG. This indicated that glucose concentration plays a key role in the shift of C2C12 cell metabolic pathways. The anaerobic metabolism of C2C12 cells was detected in HG culture with evidence of lactate production. Considerable increase in lactate production was found in the culture exposed to hypoxic environment compared to normoxic environment, suggesting a compensatory increase of anaerobic metabolism in hypoxia. The C2C12 metabolic activity in LG culture exposed to hypoxic environment was found to be much lower than normoxic environment. The introduction of higher glucose concentrations (MG and HG) resulted in elevated cellular metabolic activity in hypoxic cultures, implicating glucose as the energy source for this activity. 

There are some limitations to our study. Firstly, as mentioned in the Result Section, due to technical problem, oxygen consumption rates during hypoxia could not be measured using the oxygen biosensor plate as we did for normoxia. Secondly, Alamar Blue data were normalized to contemporaneous cell counts in order to eliminate the chance of confusing an alteration in cell number with an alteration in cell metabolism. However, the data after normalization (Figure 5) showed less consistency than that before normalization (Figure 4). This was most likely due to difficulties in detaching and counting cells in the small wells of the 96-well plate, which may be resolved using other counting strategies in the future.

## 5. Conclusions

In this study, the aerobic and anaerobic respirations in C2C12 murine skeletal myoblasts were investigated by measuring oxygen consumption, glucose consumption, lactate production, and metabolic activity in different glucose concentrations, under conditions of normoxia and hypoxia. Our findings suggest that a combination of low oxygen and low glucose damages the viability of C2C12 cells more seriously than low oxygen alone. In addition, when there is sufficient glucose C2C12 cells will respond to hypoxia by upregulating anaerobic respiration, as shown by lactate production. 

## Abbreviations

OC: Oxygen conformance
ATP: Adenosine-5′-triphosphate
atm: Atmosphere
LG: Low-glucose
MG: Medium glucose
HG: High glucose
OUR: Oxygen utilization rate
EM: Experiment media.

## References

[1] Mizock BA, Falk JL (1992). Lactic acidosis in critical illness. *Critical Care Medicine*.

[2] Paquot N, Schneiter P, Cayeux MC (1995). Effects of infused sodium lactate on glucose and energy metabolism in healthy humans. *Diabete et Metabolisme*.

[3] Saladin KS (2004). *Anatomy and Physiology: The Unity of Form and Function*.

[4] Hochachka P, Guppy M (1987). *Arrest and Control of Biological Time*.

[5] Gnaiger E (2003). Oxygen conformance of cellular respiration: a perspective of mitochondrial physiology. *Advances in Experimental Medicine and Biology*.

[6] Hogan MC, Kurdak SS, Arthur PG (1996). Effect of gradual reduction in O_2_ delivery on intracellular homeostasis in contracting skeletal muscle. *Journal of Applied Physiology*.

[7] Budinger GRS, Chandel N, Shao ZH (1996). Cellular energy utilization and supply during hypoxia in embryonic cardiac myocytes. *The American Journal of Physiology*.

[8] Subramanian RM, Chandel N, Scott Budinger GR, Schumacker PT (2007). Hypoxic conformance of metabolism in primary rat hepatocytes: a model of hepatic hibernation. *Hepatology*.

[9] Forgan LG, Forster ME (2009). Oxygen consumption and blood flow distribution in perfused skeletal muscle of chinook salmon. *Journal of Comparative Physiology B*.

[10] Andrés V, Walsh K (1996). Myogenin expression, cell cycle withdrawal, and phenotypic differentiation are temporally separable events that precede cell fusion upon myogenesis. *Journal of Cell Biology*.

[11] Shimokawa T, Kato M, Ezaki O, Hashimoto S (1998). Transcriptional regulation of muscle-specific genes during myoblast differentiation. *Biochemical and Biophysical Research Communications*.

[12] Lawson MA, Purslow PP (2000). Differentiation of myoblasts in serum-free media: effects of modified media are cell line-specific. *Cells Tissues Organs*.

[13] Delgado I, Huang X, Jones S (2003). Dynamic gene expression during the onset of myoblast differentiation in vitro. *Genomics*.

[14] Shen X, Collier JM, Hlaing M (2003). Genome-wide examination of myoblast cell cycle withdrawal during differentiation. *Developmental Dynamics*.

[15] Burattini S, Ferri R, Battistelli M, Curci R, Luchetti F, Falcieri E (2004). C2C12 murine myoblasts as a model of skeletal muscle development: Morpho-functional characterization. *European Journal of Histochemistry*.

[16] Tannu NS, Rao VK, Chaudhary RM (2004). Comparative proteomes of the proliferating C2C12 myoblasts and fully differentiated myotubes reveal the complexity of the skeletal muscle differentiation program. *Molecular and Cellular Proteomics*.

[17] Veliça P, Bunce CM (2011). A quick, simple and unbiased method to quantify C2C12 myogenic differentiation. *Muscle and Nerve*.

[18] Zhang L, Shi S, Zhang J, Zhou F, Ten Dijke P (2012). Wnt/*β*-catenin signaling changes C2C12 myoblast proliferation and differentiation by inducing Id3 expression. *Biochemical and Biophysical Research Communications*.

[19] Okano T, Matsuda T (1998). Tissue engineered skeletal muscle: preparation of highly dense, highly oriented hybrid muscular tissues. *Cell Transplantation*.

[20] Dennis RG, Kosnik PE (2000). Excitability and isometric contractile properties of mammalian skeletal muscle constructs engineered in vitro. *In Vitro Cellular and Developmental Biology*.

[21] Dennis RG, Kosnik PE, Gilbert ME, Faulkner JA (2001). Excitability and contractility of skeletal muscle engineered from primary cultures and cell lines. *The American Journal of Physiology*.

[22] Srikakulam R, Winkelmann DA (2004). Chaperone-mediated folding and assembly of myosin in striated muscle. *Journal of Cell Science*.

[23] Gawlitta D, Li W, Oomens CWJ, Baaijens FPT, Bader DL, Bouten CVC (2007). The relative contributions of compression and hypoxia to development of muscle tissue damage: an in vitro study. *Annals of Biomedical Engineering*.

[24] Lee W-Y, Cheng W-Y, Yeh Y-C (2011). Magnetically directed self-assembly of electrospun superparamagnetic fibrous bundles to form three-dimensional tissues with a highly ordered architecture. *Tissue Engineering C*.

[25] Arthur PG, Giles JJ, Wakeford CM (2000). Protein synthesis during oxygen conformance and severe hypoxia in the mouse muscle cell line C2C12. *Biochimica et Biophysica Acta*.

[26] Gawlitta D, Oomens CWJ, Bader DL, Baaijens FPT, Bouten CVC (2007). Temporal differences in the influence of ischemic factors and deformation on the metabolism of engineered skeletal muscle. *Journal of Applied Physiology*.

[27] Heywood HK, Bader DL, Lee DA (2006). Rate of oxygen consumption by isolated articular chondrocytes is sensitive to medium glucose concentration. *Journal of Cellular Physiology*.

[28] Guarino RD, Dike LE, Haq TA, Rowley JA, Pitner JB, Timmins MR (2004). Method for determining oxygen consumption rates of static cultures from microplate measurements of pericellular dissolved oxygen concentration. *Biotechnology and Bioengineering*.

